# A pipeline to further enhance quality, integrity and reusability of the NCCID clinical data

**DOI:** 10.1038/s41597-023-02340-7

**Published:** 2023-07-27

**Authors:** Anna Breger, Ian Selby, Michael Roberts, Judith Babar, Effrossyni Gkrania-Klotsas, Jacobus Preller, Lorena Escudero Sánchez, AIX-COVNET Collaboration, Sören Dittmer, Matthew Thorpe, Julian Gilbey, Anna Korhonen, Emily Jefferson, Georg Langs, Guang Yang, Xiaodan Xing, Yang Nan, Ming Li, Helmut Prosch, Jan Stanczuk, Jing Tang, Philip Teare, Mishal Patel, Marcel Wassink, Markus Holzer, Eduardo González Solares, Nicholas Walton, Pietro Liò, Tolou Shadbahr, James H. F. Rudd, John A. D. Aston, Jonathan R. Weir-McCall, Evis Sala, Carola-Bibiane Schönlieb

**Affiliations:** 1 Department of Applied Mathematics and Theoretical Physics, University of Cambridge, Cambridge, UK; 2 Center of Medical Physics and Biomedical Engineering, Medical University of Vienna, Vienna, Austria; 3 Department of Radiology, University of Cambridge, Cambridge, UK; 4 Cambridge University Hospitals NHS Trust, Cambridge, UK; 5 Department of Medicine, University of Cambridge, Cambridge, UK; 6 School of Clinical Medicine, University of Cambridge, Cambridge, UK; 7 Cancer Research UK (CRUK) Cambridge Centre, Cambridge, UK; 8 Department of Pure Mathematics and Mathematical Statistics, University of Cambridge, Cambridge, UK; 9 Department of Radiology, Royal Papworth Hospital, Cambridge, UK; 10 Advanced Radiodiagnostics Centre, Fondazione Policlinico Universitario Agostino Gemelli, Rome, Italy; 11 Università Cattolica del Sacro Cuore, Rome, Italy; 12 Department of Mathematics, University of Manchester, Manchester, UK; 13 Language Technology Laboratory, University of Cambridge, Cambridge, UK; 14 Population Health and Genomics, School of Medicine, University of Dundee, Dundee, UK; 15 Department of Biomedical Imaging and Image-guided Therapy, Computational Imaging Research Lab Medical University of Vienna, Vienna, Austria; 16 National Heart and Lung Institute, Imperial College London, London, UK; 17 Research Program in Systems Oncology, Faculty of Medicine, University of Helsinki, Helsinki, Finland; 18 Data Science & Artificial Intelligence, AstraZeneca, Cambridge, UK; 19 Clinical Pharmacology & Safety Sciences, AstraZeneca, Cambridge, UK; 20 contextflow GmbH, Vienna, Austria; 21 Institute of Astronomy, University of Cambridge, Cambridge, UK; 22 Department of Computer Science and Technology, University of Cambridge, Cambridge, UK

**Keywords:** Predictive markers, Diagnostic markers, Prognostic markers

## Abstract

The National COVID-19 Chest Imaging Database (NCCID) is a centralized UK database of thoracic imaging and corresponding clinical data. It is made available by the National Health Service Artificial Intelligence (NHS AI) Lab to support the development of machine learning tools focused on Coronavirus Disease 2019 (COVID-19). A bespoke cleaning pipeline for NCCID, developed by the NHSx, was introduced in 2021. We present an extension to the original cleaning pipeline for the clinical data of the database. It has been adjusted to correct additional systematic inconsistencies in the raw data such as patient sex, oxygen levels and date values. The most important changes will be discussed in this paper, whilst the code and further explanations are made publicly available on GitLab. The suggested cleaning will allow global users to work with more consistent data for the development of machine learning tools without being an expert. In addition, it highlights some of the challenges when working with clinical multi-center data and includes recommendations for similar future initiatives.

## Introduction

First introduced in 2020, in response to the emergence of Coronavirus Disease 2019 (COVID-19) as a global pandemic, the National COVID-19 Chest Imaging Database (NCCID)^1^ is a centralized database that contains computed tomography (CT), chest X-ray (CXR) and magnetic resonance (MR) images collected from over 20,000 patients across the UK. The aim of NCCID is to support a better understanding of the SARS-CoV-2 virus and help in the development of machine learning tools, amongst others, to enable optimized care for hospitalized patients. At participating hospitals, data was requested for all patients with a positive COVID-19 reverse transcription polymerase chain reaction (RT-PCR or simply PCR) test as well as a random sample of patients who tested negatively. The quantity of negative patients was similar to the positive cases in the first disease ‘waves’ of 2020 and 2021, but has since increased.

Requests to access the NCCID can be made online, giving access to the comprehensive clinical data available in addition to pseudonymized Digital Imaging and Communication In Medicine (DICOM) image files. A large range of clinical features (including patient demographics, past medical history, laboratory results and outcomes) are provided for patients with a positive test for COVID-19 as well as selected features for patients with a negative PCR.

A cleaning pipeline for the NCCID clinical data was published on GitHub in 2021 (https://github.com/nhsx/nccid-cleaning/tree/v0.3.0) by NHSx (which has since been incorporated into NHS England) with the database now being managed by the NHS AI Lab. We will subsequently refer to it as the NHSx pipeline. The packaged cleaning pipeline includes remapping, clipping and rescaling of features that appear to contain messy data. The data was collected during a time of high pressure and resource demands, therefore inconsistencies are expected and some systematic errors can be traced back.

Cleaning of inconsistent or defective data is of high importance in order to allow reasonable usage for diagnostic analyses and the development of high-quality machine learning tools. The urgent need for COVID-19 models to get a fast, automated prognosis for clinical triage has luckily decreased in the last year, but nevertheless the tremendous amount of collected COVID-19 related data throughout the globe reveals uniquely powerful possibilities. When systematically stored and cleaned, such huge amounts of medical data can provide an outstanding chance for the development of machine learning tools in the medical domain beyond the pandemic; E.g. to provide models that can easily be adapted to diverse diseases and/or to enhance the understanding of deep learning related approaches.

As mentioned above, a major drawback of data collection during the COVID-19 pandemic was the scarcity of resources (e.g. time), resulting in inconsistent and out of range data. This complicated the use of multi-center datasets for non-medical experts as judging the plausibility of medical values requires expert knowledge. With our pipeline, we aim to contribute to the sustainability of data collection and to enable non-experts to work with the NCCID clinical data. This work will inform future multi-center projects, akin to NCCID, by providing examples of the challenges that are encountered when using such medical data as well as giving specific recommendations for dataset curators and developers.

The outline of the paper is as follows. In Section 2 we state the most important improvements obtained when using our pipeline in comparison to the raw data or that cleaned by the NHSx pipeline. In Section 3 the importance of these results are discussed, whereas in Section 4 the methods used to identify the issues and implement the cleaning functions are explained. The data is available online upon request at the website of the NHS AI Lab (https://nhsx.github.io/covid-chest-imaging-database/) and our cleaning pipeline (including the required components from the NHSx pipeline) is available open-source on GitLab https://gitlab.developers.cam.ac.uk/maths/cia/covid-19-projects/nccidxclean. Information about every function in the pipeline is provided in detail in our online documentation (https://maths.uniofcam.dev/cia/covid-19-projects/nccidxclean/).

## Results

In this section we provide examples of the improvements that are achieved when using our cleaning pipeline in comparison to the raw data and the NHSx pipeline. In total, at the time of final development (November 2022), 21,253 patient cases were available from 25 hospital groups in England and Wales. 6,931 patients at 23 of the hospital groups had a positive PCR test with 2 organisations having only submitted patients who tested negative. We abbreviate the names of the hospitals following the key in Table 1 to increase the readability of the manuscript.

**Table 1:** Codes used to represent the hospitals and the 23 NHS Trusts (England)/Health Boards (Wales).

Code	Hospital(s)	Submitting Center
A	Ashford and St Peter’s Hospital	Ashford and St Peters Hospitals NHS Foundation Trust
B	Unknown	Betsi Cadwaladr University Health Board
C	Princess Royal Hospital	Brighton and Sussex University Hospitals NHS Trust
D	Royal Sussex County Hospital	Brighton and Sussex University Hospitals NHS Trust
E	Unknown	Brighton and Sussex University Hospitals NHS Trust
F	Prince Charles Hospital	Cwm Taf Morgannwg University Health Board
G	Royal Glamorgan Hospital	Cwm Taf Morgannwg University Health Board
H	Ysbyty Cwm Cynon	Cwm Taf Morgannwg University Health Board
I	George Eliot Hospital	George Eliot Hospital NHS Trust
J	Basingstoke and North Hampshire Hospital	Hampshire Hospitals NHS Foundation Trust
K	Royal Hampshire County Hospital	Hampshire Hospitals NHS Foundation Trust
L	Charing Cross Hospital	Imperial College Healthcare NHS Trust
M	Hammersmith Hospital	Imperial College Healthcare NHS Trust
N	St. Mary’s Hospital	Imperial College Healthcare NHS Trust
O	Unknown	Leeds Teaching Hospitals NHS Trust
P	Liverpool Heart and Chest Hospital	Liverpool Heart and Chest NHS Foundation Trust
Q	Ealing Hospital	London North West University Healthcare NHS Trust
R	Northwick Park Hospital	London North West University Healthcare NHS Trust
S	Norfolk & Norwich University Hospital	Norfolk and Norwich University Hospitals NHS Foundation Trust
T	Unknown	Oxford University Hospitals NHS Foundation Trust
U	Unknown	Royal Cornwall Hospitals NHS Trust
V	Royal Surrey County Hospital	Royal Surrey NHS Foundation Trust
W	Royal United Hospital	Royal United Hospitals Bath NHS Foundation Trust
X	Unknown	Sandwell and West Birmingham Hospitals NHS Trust
Y	Sheffield Children’s Hospital	Sheffield Childrens NHS Foundation Trust
Z	Unknown	Sheffield Teaching Hospitals NHS Foundation Trust
*α*	St George’s Hospital	St Georges University Hospitals NHS Foundation Trust
*β*	Musgrove Park Hospital	Taunton and Somerset NHS Foundation Trust
*γ*	The Walton Centre	The Walton Centre NHS Foundation Trust
*δ*	Unknown	University Hospitals of Leicester NHS Trust
*ε*	West Suffolk Hospital	West Suffolk NHS Foundation Trust

The ‘Submitting Center’ in the NCCID corresponds to the organization submitting the data (the NHS Trust or Health Board), which may operate multiple hospitals. When possible, individual hospitals were used in the analysis to allow more detailed insights such as identification of inconsistencies between hospitals of the same submitting center.

Microsoft Excel spreadsheets were used for the collection of the clinical data, filled in by each submitting center and then uploaded via a web portal. The templates are available online in their original form (https://medphys.royalsurrey.nhs.uk/nccid/guidance.php), containing 68 data fields for positive patients and 7 for negative patients. Consequently, the vast majority of the fields only apply to patients who had a positive COVID-19 diagnosis and a huge amount of the data will always be missing by design when the full data is aggregated. To combat this, we will only discuss and show the results for the positive cohort from this point onward. In the following we will refer to the individual data fields as features.

**Fig. 1:**
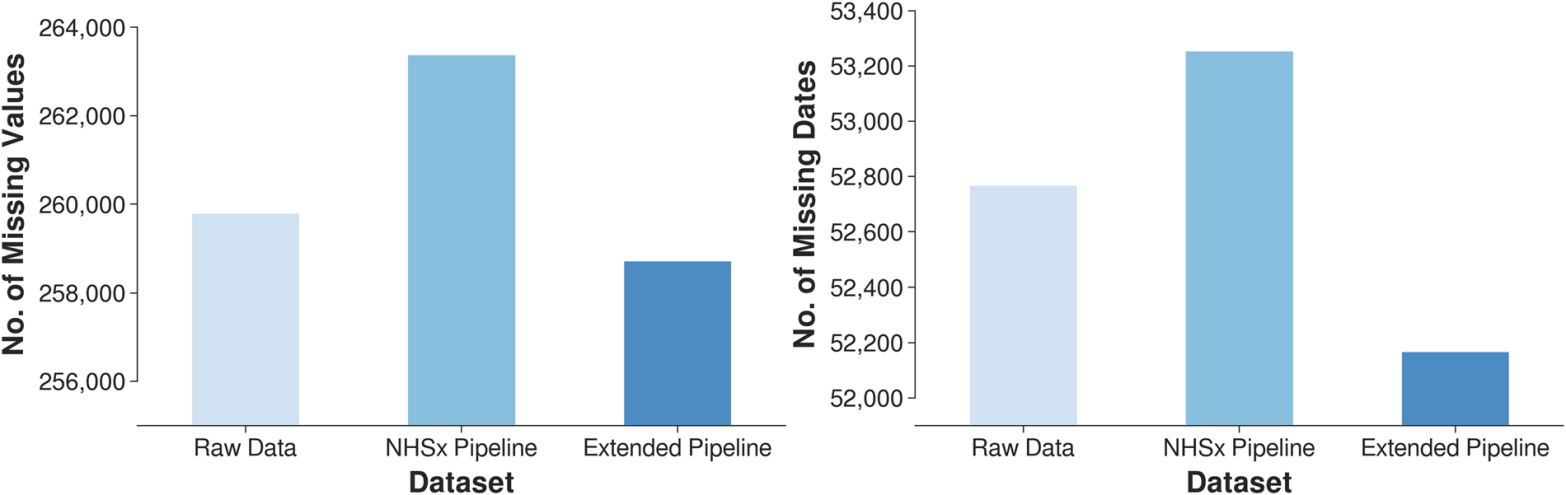
Number of missing values (left) and dates (right) in the raw data, after cleaning with the NHSx and the extended pipeline.

The development of our cleaning pipeline was guided by structural inconsistencies, e.g. date formatting errors and implausible biomedical values. The application of the pipeline leads to a reduced number of missing values in comparison to the raw and the NHSx cleaned data, see Fig. 1. The correction of all features is described in detail in the documentation available on GitLab and in this paper we focus on the description of 10 representative examples. The underlying methods will be discussed in detail in Section 3.

### Categorical features

**Fig. 2:**
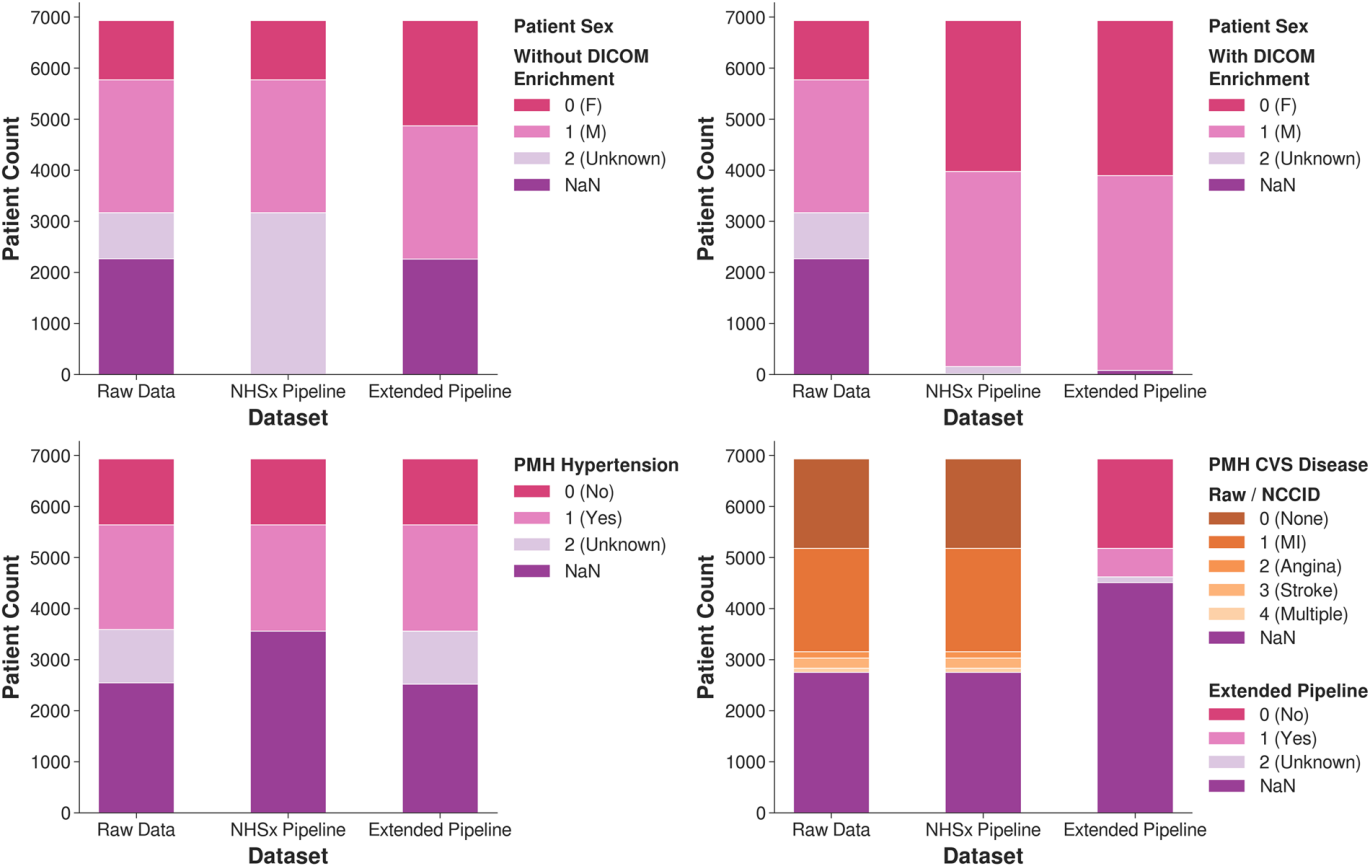
Adjustment of the categorical features *Sex*, *PMH CVS Diseases* and *PMH Hypertension*. ‘NaN’ refers to missing data values.

Some systematic inconsistencies appeared in the categorical features including the handling of ‘Unknown’ versus blank entries, categorical confusions such as ‘F/M’ or ‘1/2’ versus ‘0/1’ for *Sex*, and misspellings such as ‘1es’ instead of ‘1’. See Fig. 2 for our corrections regarding *Sex*, past medical history *(PMH) of Cardiovascular (CVS) Disease* and *PMH Hypertension*.

Without using individualized DICOM information, the number of patients classified as female increased from 1,162 to 2,064 (78% increase), when compared to the NHSx pipeline and the number of males remained 2,064. With additional updates from DICOM metadata, the number of patients classified as either male or female increased from 6,776 to 6,853 (1.1% increase).

For *PMH Hypertension*, 1,038 ‘Unknown’ values have been retained in comparison to the NHSx pipeline that removed them, leaving them blank. Distinguishing between unknown and missing values (here referred to as ‘NaN’) is important as they represent two distinct types of data that can have different implications for analysis and interpretation. ‘NaN’ values refer to data that is absent or unavailable, for reasons such as data entry errors, data loss, or data that was not collected. ‘Unknown’ values refer to data where the value is unknown or undefined due to the nature of the data or the data collection process. We found that the ‘Unknown’ values in the *PMH Hypertension* feature showed quite different statistical behavior compared to the original ‘NaN’ values, in particular

- 77% of patients with ‘NaN’ were male, compared to 62% of ‘Unknowns’,
- 34% of patients with ‘NaN’ had a history of lung disease, compared to 13% of ‘Unknowns’,
- 37% of patients with ‘NaN’ had a history of *Chronic Kidney Disease (CKD)*, compared to 10% of ‘Unknowns’,
- 3% of patients with ‘NaN’ value required intubation, compared to 10% of ‘Unknowns’.

The number of patients categorized as having *PMH CVS Disease* fell from 58% to 23%. This is a result of disregarding all values for hospital X within this feature since‘1’ was exclusively entered, suggesting that all of their 1,872 patients had previously suffered a myocardial infarction. Additionally, the *PMH CVS Disease* (included in Fig. 2) and *PMH Lung Disease* features have been collapsed to include only ‘Yes’, ‘No’ and ‘Unknown’ values. Our reasoning for this loss of information is discussed in the Methods section. However, the user can opt to retain the original categories by changing an input parameter when running the extended pipeline. Beyond these examples, similar adjustments have been applied to *Ethnicity* and all other categorical PMH and medication features.

### Dates

Due to inconsistencies in use of UK versus US date format, there appeared confusion from the data entries themselves compounded by those resulting from file formatting. Using the NHSx pipeline, there were an additional 488 missing date values after cleaning when compared to the raw data. With our extended pipeline, the number of missing dates fell by 1,087 (3.6%) when compared to the NHSx pipeline and 699 (2.3%) when compared to the raw data, see Fig. 1.

**Fig. 3:**
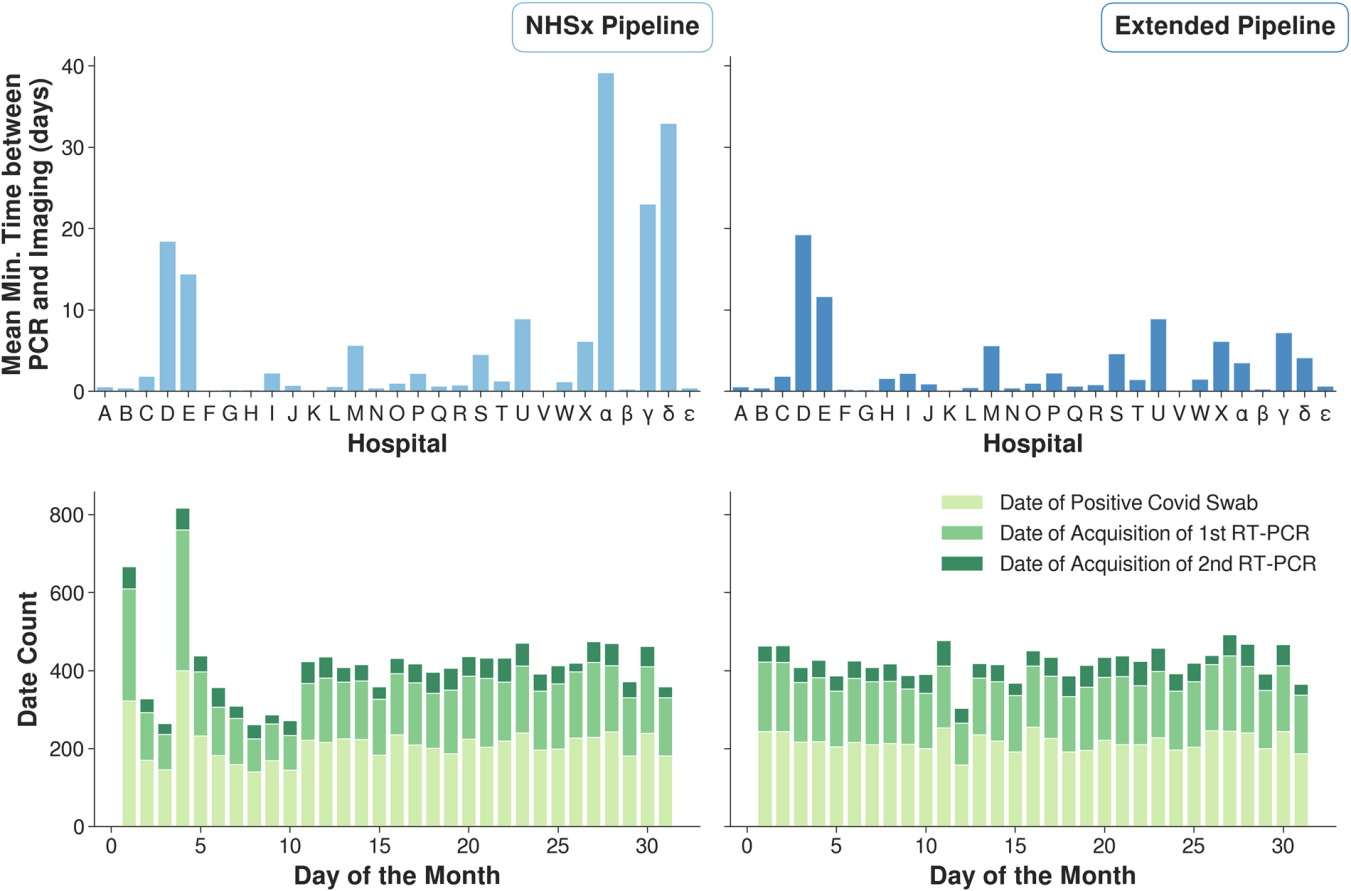
(Top) Mean time of smallest time lag between a PCR test and corresponding imaging for each patient. (Bottom) Distribution of days throughout the month for the collected dates.

For three of the date features (*Date of Admission*, *Date of Acquisition of 1st RT-PCR* and *Date of Positive Covid Swab*) which were expected in US format (mm/dd/yyyy), 3 hospitals (*α*, *γ*, *δ*) were found to have submitted dates in UK format (dd/mm/yyyy). In the original cleaning pipeline, 67% of affected dates were still processed correctly, but in 33% of the remaining cases the error went undetected and 1,664 incorrect dates were obtained. In Fig. 3 (top) the amount of time between the affected PCR date and the closest imaging date has been computed for each patient. When applying our pipeline it can be observed that high time gaps were eliminated at the affected hospitals. In Fig. 3 (bottom) the number of values each day throughout the month is shown for the three date related features. The spikes on days 1 and 4 in the plot of the NHSx cleaned data (left) have been removed.

### Numerical features

**Fig. 4:**
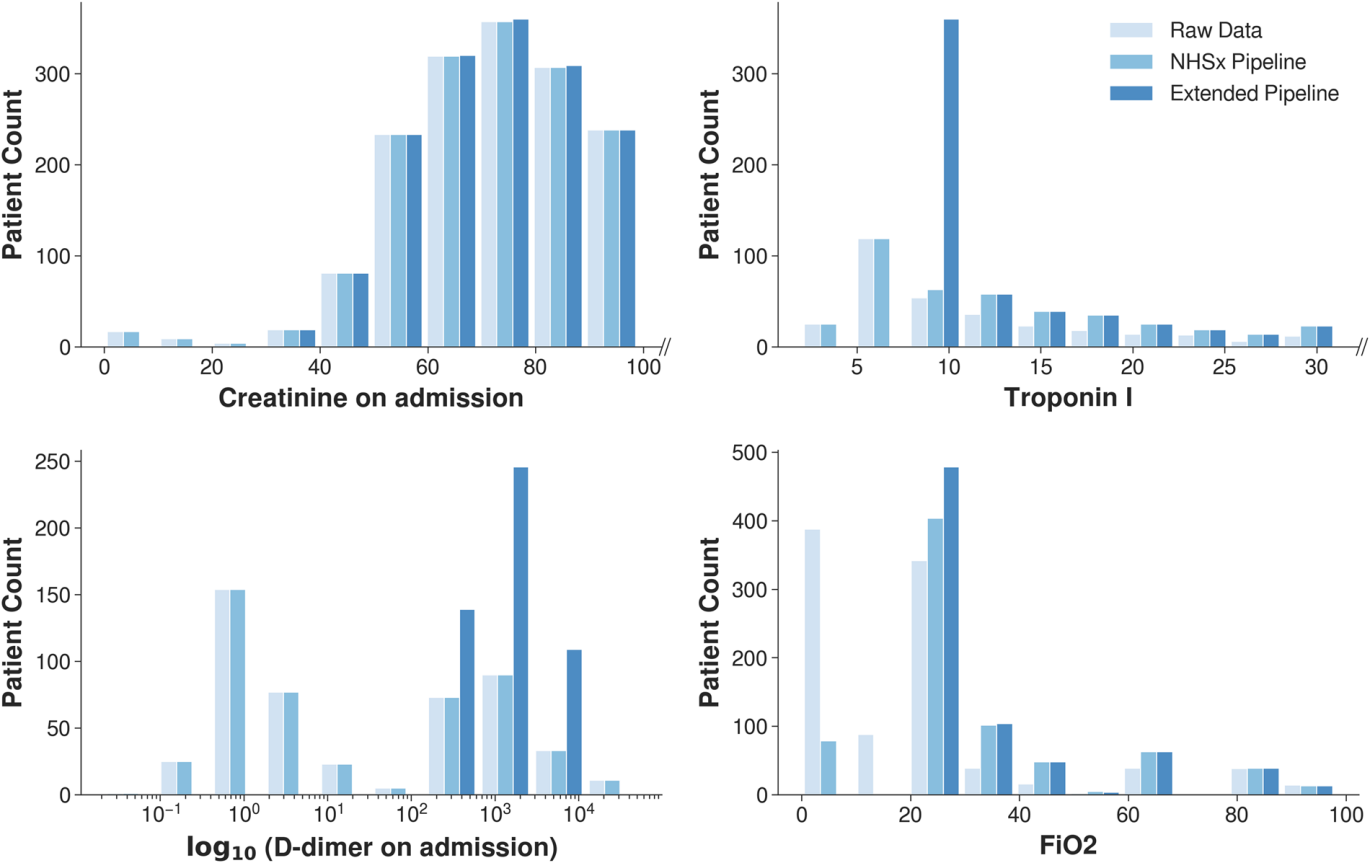
Adjustment of the blood test features and administered oxygen levels.

The features *Creatinine*, *Troponin* and *D-dimer* obtained from blood tests and the fraction of inspired oxygen parameter *FiO*_2_ have been corrected according to biological and technical threshold values as well as unit inconsistencies, see Fig. 4 for the comparison of the original and updated numerical values. Around 6% of *Troponin I* values that would have been lost in the original pipeline, as they contained a less than symbol (‘ < ’), were retained, whilst 186 values (26%) were truncated to a value of 10. *Creatinine* values for 22 samples (0.87%) were thresholded and the units were corrected for 8 (0.32%) values. As we see in Fig. 4, there are no longer any *FiO*_2_ values below 21% and the distribution of the *D-dimer* results now shows a unimodal distribution. The spike in *FiO*_2_ just above zero likely represented values being expressed as a decimal rather than a percentage, which have now been corrected.

**Fig. 5:**
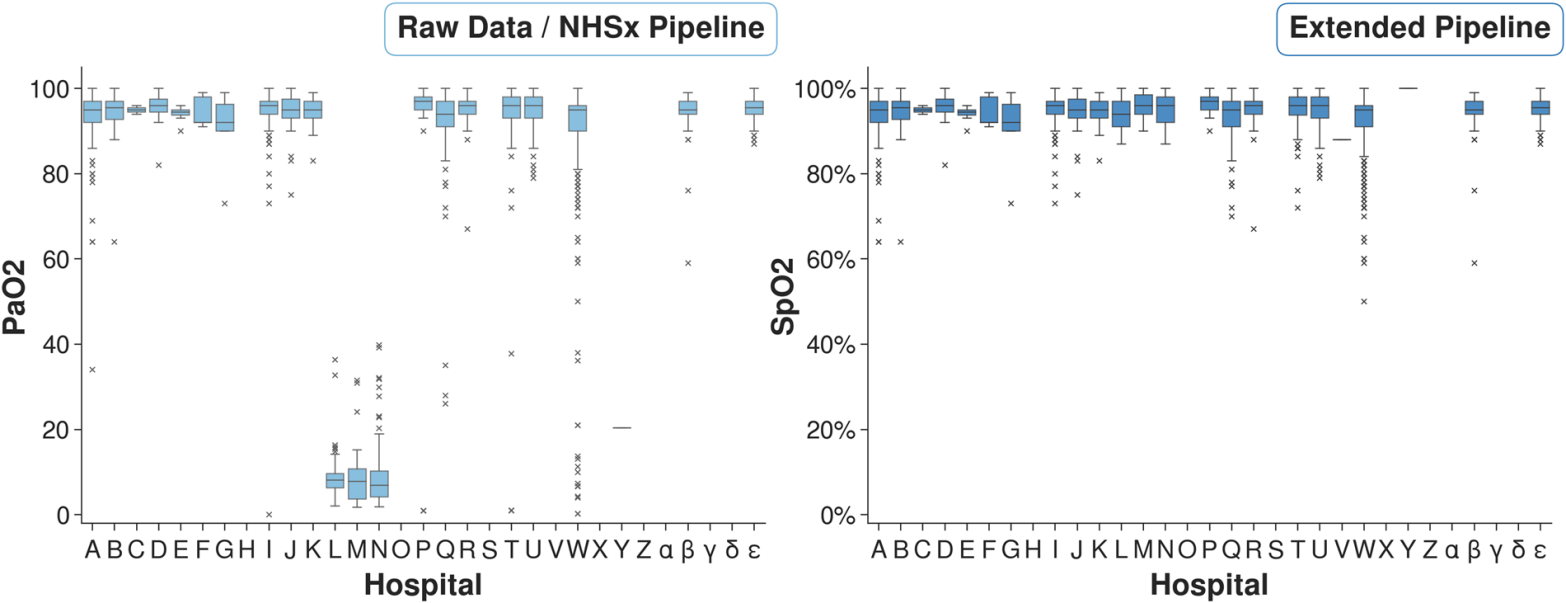
Box-plots of original *PaO2* values (left) and our *SpO2* output demonstrating more consistent values (right).

For patient oxygen levels (originally the *PaO2* feature), 4 of hospitals submitted partial pressures of oxygen (PaO_2_), 17 provided oxygen saturations (SpO_2_), and 1 provided a mixture of both. The PaO_2_ values have been converted to the more commonly measured SpO_2_, including a correction of units, see Fig. 5. 149 values in the new *SpO2* feature were inferred from likely PaO_2_ values (8.5% of completed entries).

## Discussion

Due to the unprecedented pressure on resources in hospitals during the data collection period of the NCCID, many inconsistencies and systematic errors are found in the data. Some of these issues can be traced back and many have been corrected by the original NHSx pipeline. However, a number of important issues still remain which can reduce the relevance and generalizability of subsequently developed models, as we will discuss shortly. These have been identified and corrected by our extended pipeline where possible. Here, we discuss some of the key issues, their consequences and the impact of solving them. Although specific to NCCID, similar issues will be applicable to other such collaborative datasets. As such, we will subsequently provide recommendations of how data curation and cleaning may be improved in similar future projects.

### Fairness, reproducibility and interpretability

Understanding of the data used to train machine learning models is fundamental to ensuring ethical and fair algorithms. This is of paramount importance in the medical domain since outputs may directly influence clinical decision making and patient care. As stated in^2^, data “truthfulness” includes understanding of how complete and detailed given data are, what information they contain, how accurately they reflect the true physical situation as well as measures of variance and bias. A certain level of bias is unavoidable in any dataset; however, its impact may be mitigated by gaining a thorough understanding of the data and any potential sources of bias^3,4^. For example, if there is selection bias in the data collection or model development, it may be necessary to test the model on additional data to ensure that it is adequately evaluated on all groups prior to deployment^3,5^. Major generalization failures of machine learning models have been reported, especially when relying on age and sex^6,7^. Amongst others, diagnostic COVID-19 models have been shown to under-diagnose the female and younger patients^8^ when they are under-represented in the training data^9^.

All papers published in the first year of the COVID-19 pandemic failed to satisfy sufficient criteria regarding reproducibility and interpretability for clinical use despite the urgent need of tools to assist clinicians^4,10^. Although this was partly a consequence of hidden biases within the data, it is further compounded by a failure to adequately perform data cleaning and/or standardization as well as describing the models in sufficient detail^11^.

### Categorical features

#### Sex

In addition to the many ‘NaN’ and ‘Unknown’ values in the raw data, the distribution of sex was far from the ratio in the national population (51:49 for F:M in the UK^12^). Such bias in the data can yield impactful, misleading conclusions when used for downstream analysis. We can observe that our established distribution of ‘F’ versus ‘M’ corresponds to the expected ratio of sex in contrast to the original data due to recovery of many ‘F’ values - even when not explicitly extracting information from DICOM metadata (Fig. 2). With our cleaning the data is more representative to the population, selection bias is reduced and subsequent models will be more reliable, with results more likely to be reproducible on local data.

Although the DICOM metadata is theoretically available on request, it cannot be assumed that each user of the clinical data would store the corresponding images with DICOM headers (as they require a large amount of storage capacity), whilst some users may only utilise the clinical part of the database. Therefore, it is important to implement a solution that does not need information from user stored DICOM files. This was possible in our pipeline by identifying that one hospital systematically inserted illegitimate values, see Section 4 for more details. When enriching the data with the DICOMs, the NHSx pipeline yielded still yielded a slightly higher number of ‘NaN’/‘Unknown values’.

#### PMH CVS Disease

A high proportion of implausible data values is very likely to result in misleading and biased results when incorporated into any learning model. The increase in ‘NaN’ values is undesirable here, but unfortunately necessary, since one hospital (X) inserted the same value (corresponding to myocardial infarction or more commonly, heart attack) for all of their patients, making up 45% of non-missing values in that category, see Fig. 7. We concluded that this is implausible and therefore one third of all *PMH CVS Disease* values had to be removed.

#### PMH Hypertension

We use this feature as an example of one major change that we applied for all categorical features, namely preserving the ‘Unknown’ category in comparison to the NHSx pipeline which removed all of these values, placing them in the category ‘NaN’. We believe that there are cases in which it is important to distinguish between values that have not been inserted at all and values that have been explicitly inserted as ‘Unknown’. Given that the ‘NaN’ patients were more likely to be older, male, have a history of lung and kidney disease, and to have been intubated, we believe that this demonstrates that ‘Unknown’ and ‘NaN’ are not necessarily equivalent values. In addition, we found that in the other categorical fields, despite having very similar numbers of ‘positive’ entries, patients with ‘NaN’ were less likely to have negative ‘0’ entries. This suggests that in some cases, rather than enter a negative value, the data curator left the field empty thinking that this was equivalent to a ‘0’.

### Dates

Date error corrections are of high importance since these labels are also used as annotations to train automated prediction tasks (e.g. prediction of death within 48 h of admission) related to COVID-19. A high number of false dates will lead to the development of machine learning tools that are biased towards a false prediction and will fail to generalize well when applied to external data.

It is not obvious how to evaluate the correctness of dates when the day and month is simply swapped. In order to get a proper estimation, we use two indicators for sense-checking. Firstly, we compute the minimum time between a PCR test and the corresponding closest imaging date, and secondly, we analyze the distribution of days throughout the month (Fig. 3). The results obtained, regarding time between a PCR test and the closest imaging date, now correspond to what one would expect, since the imaging data primarily were acquired close to the date when a positive PCR test was obtained. Secondly, the uniform distribution of days throughout the month now coincides more with what one would expect.

### Numerical features

**Creatinine,** **Troponin I** and **FiO**_**2**_ values have been clipped and truncated according to their plausible ranges and identified laboratory detection limits (Figs. 4, 5). Allowing implausible values could yield wrong conclusions in subsequent analyses. Additionally, allowing results from different laboratories to have different possible minima and maxima to others, risks associated bias and over-fitting of algorithms. However, it is not only the minima and maxima that may differ but also the actual results themselves. The same blood sample tested on different analysers may give slightly different results and in some instances it may be more useful to provide an indicator of the deviation from normal e.g. the standard deviation, rather than the absolute numbers. Some tests also might have had different methodologies even within the same hospital, e.g. some emergency departments use rapid testing methods with more formal laboratory tests being performed later. This can result in different results, units, sensitivities, and reference ranges, and subsequently it may be unclear which value/type of test has been used for the research data. This is particularly important in the case of Troponin, where there are multiple different types of test, including not only the usual tests for Troponins I and T, but also their equivalent high-sensitivity tests. Although we have used these three tests as exemplars, it should be noted that such limitations apply to any laboratory test, including D-dimer and PaO_2_ below (Figs. 4, 5).

#### D-dimer

We observe a huge change in values by remapping all values to a consistent standard unit (ng/mL FEU). Particularly small values are affected by this change, resulting in an expected unimodal distribution.

#### PaO_2_

This feature was interpreted differently between the data providers, with some entering a PaO_2_ from an arterial blood gas and others an SpO_2_ from a saturation probe. Fortunately, given the range of likely values and the characteristics of the measurements, it was possible to distinguish between them, as demonstrated in Fig. 5 (left). Instead of the less commonly collected PaO_2_ value, our cleaning pipeline now returns a new *SpO2* feature, with values inferred from *PaO2* where necessary (more details are included in the meth section). We decided to impute these values to harmonize the oxygen data as much as possible and reduce the risk of informative missingness^13^. The latter is particularly important in this context as the presence or absence of an arterial blood gas result (*PaO2*) may be used by the model as a surrogate for the clinician’s belief of the severity of the patient’s condition. This addresses a major inconsistency error which introduced a bias between hospitals. For subsequent downstream tasks related to chest diseases (including COVID-19), the features referring to oxygen are of high importance^14^ and incorrect value interpretations could lead to impactful wrong conclusions in these tasks.

### Recommendations

The NCCID is a highly notable initiative in which 25 NHS organizations have shared pseudo-anonymized data for the development of AI for the benefit of patients in a time of need. Such projects yield great potential to exploit collaborations between organisations as COVID-19 becomes less of a dominant concern. Given that the challenges we experienced with NCCID are not unique to this dataset, we now provide recommendations for those designing the curation of such databases as well as to the developers of the models. In many respects, we were unprepared for COVID-19 and it is imperative that we learn lessons from the recent pandemic to allow us to better respond if required in the future.

### Recommendations for dataset curators

#### Develop or utilize existing data standards

We suggest to develop organisational data collection and reporting standards or utilise existing systems, such as the Study Data Tabulation Model (SDTM, https://www.cdisc.org/standards/foundational/sdtm) or Systematized Nomenclature of Medicine Clinical Terms (SNOMED CT, https://www.snomed.org/) to maximise reporting consistency between sites and interoperability, e.g. by ensuring consistent measurement units and definitions. Such frameworks would facilitate pandemic preparedness by reducing the need for complex data cleaning and allow for rapid, widespread data use. Furthermore, they could be utilized for a range of projects, and would ideally be shared with partner organisations to either keep consistent standards or develop a generic way to map the data when shared between sites. In addition, consideration should be given to whether the chosen data standard does not conflate any features, such as *Sex*/*Gender* and *SpO*_2_/*PaO*_2_, but state them as separately.

#### Incorporate data validation

Implement a system that immediately checks the inserted data and flags possible errors. This might consist of a script on data submission or simply cell validation within Microsoft Excel. When combined with detailed data standards, this will solve many of the issues reported in this paper and facilitate fast access to clean data, essential to allow for AI tools to be efficiently and effectively developed in future pandemics.

#### Share technical information

Ensure each data provider supplies sufficient detail regarding the test methodology. For example, local reference ranges should be included to understand what is “normal” at that site, as these may vary between laboratories and hospitals. In addition, collection of the equipment detection ranges/limits will allow practitioners to evaluate and reduce overfitting of the data to particular sites by adjusting the data accordingly (e.g. using the deviation from normal rather than the actual result).

#### Prepare preemptive data sharing agreements

Advanced planning for the potential of future pandemics can mitigate the issues associated with the urgency to assemble and curate clinical data when resources are under strain. Ideally, data sharing initiatives, which have been accelerated due to necessity^15^, should be maintained and enhanced.

#### Offer workshops to train local experts

Linking into data standards and preemptive planning, workshops to educate those responsible for curating the data will improve the quality of data collection and facilitate faster access. For example, the number of missing values might be reduced if the data curator were aware of the importance of always inserting a value rather than only completing the cells where there is an abnormal result (e.g. putting the entry in *FiO*_2_ equal to 21% should the patient not be on oxygen, rather than leaving the field blank). Such workshops should be performed in anticipation of future emergencies rather than after the outbreak has begun. In general, we would advise, if possible, to not require data collection from essential clinical personnel, but from those with the time, resources and background (e.g. students involved in medical data science).

#### Exploit electronic health records (EHR)

Data curation will become easier with increased adoption of EHR systems since these fundamentally include certain data standards^16^. Sites with EHR systems should be prioritised for inclusion in data collection initiatives, as they likely offer rapid and more efficient data collection. Code to download and process data for submission to the shared database should be distributed across sites to ensure consistent data collection, maximise efficiency and reduce resource burdens. Once again, contributing sites and data curation methods should be preemptively determined and prepared.

### Recommendations for developers

#### Use checklists

Developers should be familiar with checklists such as CODE-EHR^17^ and the IJMEDI checklist for assessment of medical AI^18^ ensure all necessary steps are taken and documented when preparing clinical data to maximise model quality, generalizability and reproducibility. Similarly for the associated imaging, when developing DL models the Checklist for AI in Medical Imaging (CLAIM)^19^ should be utilised. Ensuring that we, as a community, maintain high standards will facilitate high quality model development when it is most

#### Perform detailed and collaborative exploratory data analysis (EDA)

Medical data is complex and variable between sites. Adequate EDA is fundamental to ensure a comprehensive understanding of the data and to allow for hidden biases, such as the ones highlighted in this paper, to be identified for model development. If directly performed by a non-expert, this should be thoroughly reviewed by someone with the relevant medical expertise. Although not replacing the need for in-depth analysis, automated EDA solutions such as DataPrep^20^, are available to assist. EDA best practice should be shared to enable fast, yet high quality development of models in future pandemics.

#### Share pre-processing pipelines and techniques

Transparency of data pre-processing increases reproducibility and allows enhanced understanding of the data. Publishing and/or using open-source data cleaning and standardization pipelines, allows non-expert developers access to clean data, resulting in the development of more generalizable and trustworthy models. By working as a community, we can ensure that we maximise preparedness for future emergencies. Moreover, downstream users can be confident that the data has been cleaned to a high quality. Furthermore, reviewers and regulators can fully understand and examine the cleaning process for any pitfalls.

### Study limitations

We have developed the methods within the pipeline based on the data collected until February 2022. The NCCID repository is updated with new data regularly. We cannot know whether more recent data, especially when obtained from new collection sites, contain unexpected inconsistencies/issues that have not been tackled by our pipeline. Nevertheless, repeating issues should be taken care of also for new data by our pipeline and warnings will be generated when data from new centers is encountered.

Many systematic errors have been identified in our analyses, nevertheless, not all error sources could be traced back/reversed (e.g. typographical errors) and there remains a significant number of missing values, with varying levels of completeness between locations. For example, some hospitals completed just one categorical value in all of their patient entries (*Sex*) and in addition, submitted incomplete DICOM metadata following strict anonymization. Here, we focused on the cleaning of provided entries, the application of imputation models^21^ would allow to increase the number of the available values further.

In addition to missing values and new centers, there are several limitations due to missing information which cannot be solved by pre-processing. Firstly, we split the data by hospital to provide as detailed analyses between locations as possible; however, split hospital information was not provided by all submitting centers. As a result, in some cases when we discuss hospitals, we may be talking about a collection of hospitals run by one organisation and this may result in some persistent unseen bias between hospitals obtained from those submitting centers.

Compounding bias between centers, no laboratory detection or reference ranges have been provided. Although it has been possible to identify detection limits in some cases, methodologies and reference ranges can vary between laboratories and results are not necessarily equivalent^22^. Moreover, the laboratory results included are only those on admission to hospital. In some cases, these results do not correspond to their COVID-19 illness and, unfortunately, this information is not provided. For example, some patients were admitted months before they had a positive RT-PCR test suggesting hospital-acquired disease. Furthermore, there is no data on the end-of-life treatment, treatment escalation plans or DNA CPR (Do Not Attempt Cardiopulmonary Resuscitation) orders. Affected patients will not receive the same treatment as other patients (e.g. intubation and ITU admission) and will have very different prognoses. Similarly, there is no vaccination data which has significantly changed the clinical scenario and there is no additional diagnoses or cause of death, i.e. whether a patient died ‘from’ COVID-19 or ‘with’ the disease, which is an important distinction when considering a prognostic model.

In addition, we decided to impute *SpO2* values from *PaO2* to reduce missingness, using the formula described in Gadrey *et al*.^23^, however this remains an estimate. SpO_2_ refers the saturation of haemoglobin (in the red blood cells) with oxygen, whereas the PaO_2_ refers to oxygen dissolved in plasma (i.e. SpO_2_ refers to an intracellular compartment and PaO_2_ to an extracellular compartment)^24^. Such a conversion uses the principles of the oxygen dissociation curve, albeit tailored to sepsis^23^. This sigmoidal curve can be shifted by a variety of factors including acid-base balance and temperature, which influence the relationship between SpO_2_ and PaO_2_ significantly. As a consequence, critically unwell patients may have unpredictable relationships between their SpO_2_ and PaO_2_ measurements. It is therefore plausible that an imputation strategy which works at population level would be unreliable for those patients who are critically ill. Therefore, we have included the option to retain the original *PaO2* and *SpO2* features when running the pipeline, giving the user autonomy to apply other techniques or imputation methods to handle the missing values.

Furthermore, it should be noted that PaO_2_ results themselves can present challenges for algorithms. Many hospitals routinely perform blood gas analysis on venous blood (usually at admission) or an intended arterial sample may accidentally be obtained from a neighbouring vein. Venous results will often still provide a result labelled as PaO_2_ even though that should only refer to arterial blood. As both appear in the patient’s records, non-trackable transcription errors in the data may have occurred. Although a rules-based approach has been employed to clip likely venous results, it can be challenging to differentiate arterial and non-arterial blood gas results. An additional complication is that there is a third measurement which we have not considered, the *arterial oxygen saturation* (SaO_2_) of haemoglobin, sometimes calculated on a point-of-care analyser alongside PaO_2_. This may contribute additional noise to the *SpO2*/*PaO2* values as SaO_2_ should be closer to the “true” haemoglobin oxygen saturation than SpO_2_ and is less prone to bias. SpO_2_ is influenced by multiple factors including the use of vasopressors, poor peripheral perfusion, and hand/body temperature can result in misleading results in critically unwell patients^25^. Specifically, it is of concern that some SpO_2_ monitors may give a false high reading in patients with darker skin pigmentation^25,26^. This is evident in Fig. 5, where some of the outlier SpO2 values (particularly hospital W) still seems implausibly low. Such low values would represent peri-arrest, but may be more likely associated with poor oximetry signal acquisition.

Finally, the NCCID provides data on patient *Sex*, and we have discussed this feature at length. However, *Gender* (including gender reassignment) has not been collected and there is a risk that the two have been conflated. Evagora-Campbell *et al*.^6^ make the case that both, *Sex* and *Gender*, are important in COVID-19 with socially constructed gender norms affecting access to COVID-19 prevention, testing and treatment.

## Methods

**Fig. 6:**
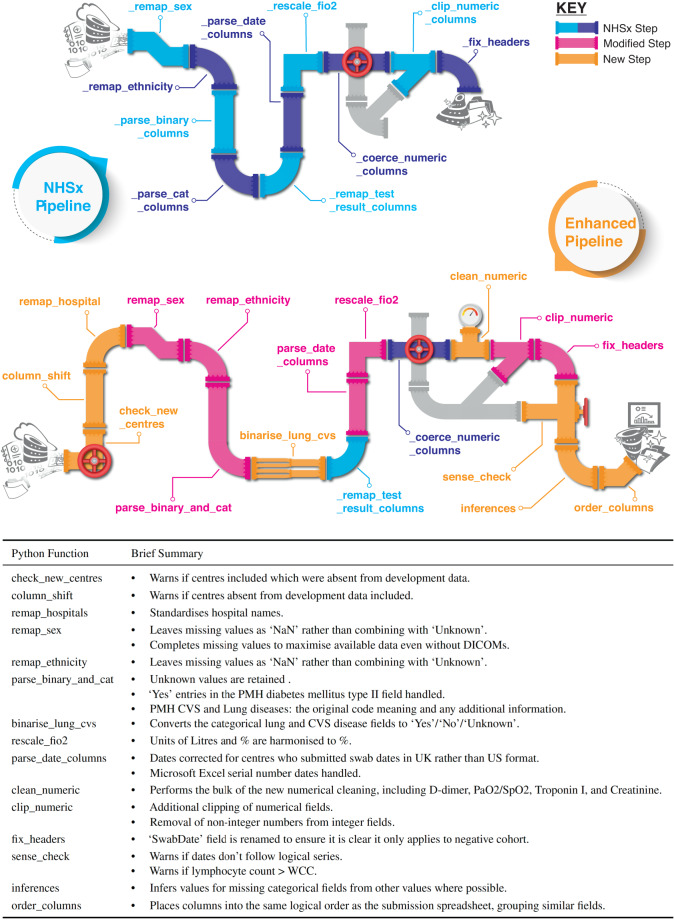
The Python functions forming the NHSx and our novel extended cleaning pipeline with brief summary of each novel and/or amended function in the extended version.

The tools developed and described in this paper are based upon extensive exploratory data analysis and our experience of using the NCCID data to develop machine learning models for COVID-19^27,28^. Given there are 68 features for the cohort with positive PCR tests, in this paper we have focused on 10 examples which best reflect our methodology and the outcomes. All changes regarding other features are described in the code documentation. Figure 6 graphically summarizes the modifications made to the NHSx pipeline.

**Fig. 7:**
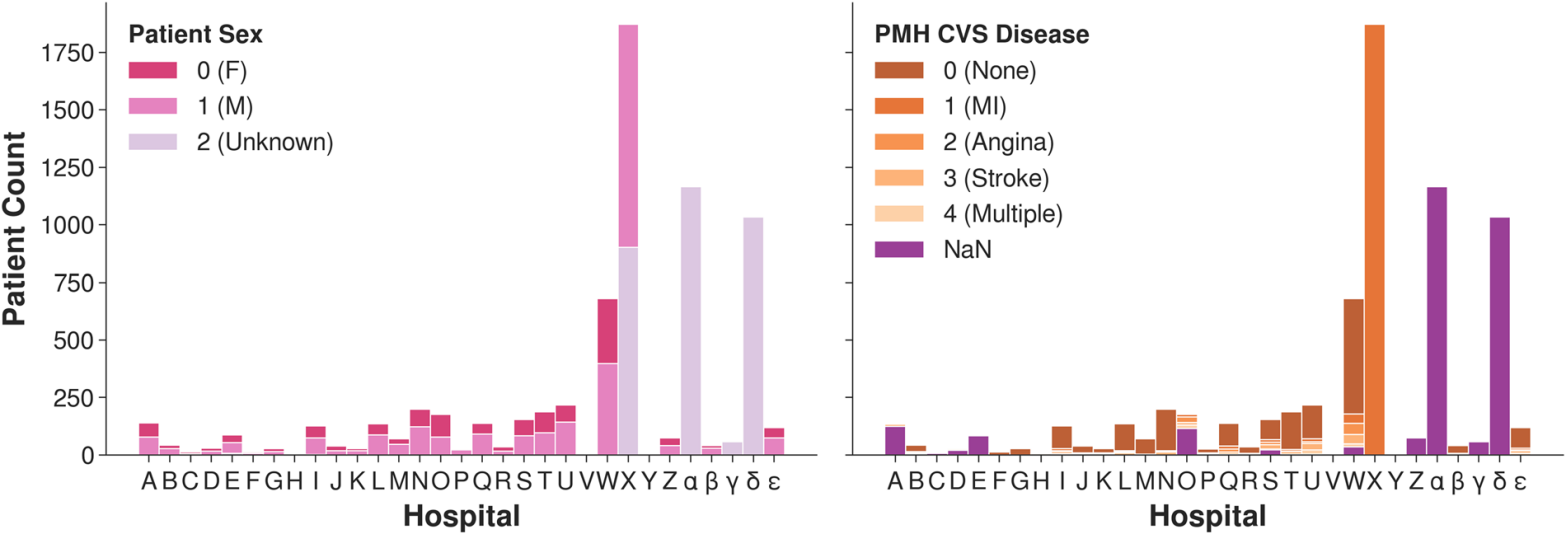
Output of NHSx cleaning pipeline for *Sex* (left) and *PMH CVS Disease* (right) per hospital.

### Categorical features

#### Sex

Exploratory data analysis as demonstrated in Fig. 7 (left), showed that hospital X had entered ‘1’ and ‘2’ rather than ‘0’ (female) and ‘1’ (male) as requested in the original spreadsheets^29^. Based on a manual comparison of a sample to the corresponding DICOM headers we were able to re-establish the meaning and corrected ‘1’ to male and ‘2’ to female systematically for all data points in that hospital. This was confirmed through review of a sample of chest X-rays by a clinical expert, using the presence of breast tissue as one indicator for the female sex. In comparison, the NHSx cleaning pipeline would set the values of ‘2’ to ‘Unknown’, only later correcting these values if the data is enriched with the DICOM metadata from the chest X-rays. Therefore, if the imaging data is not downloaded and stored alongside the clinical data, the correction is not applied. We utilised the DICOM metadata only for the development of the cleaning pipeline and the final application of the cleaning pipeline does not require it, i.e. we provide the users access to this additional sex data without necessarily downloading the imaging/DICOM files. Unfortunately, for 78 patients at hospitals *α*, *γ* and *δ*, no values were entered and they also could not be recovered from the DICOM.

#### PMH CVS Disease

Similar analysis to that of *Sex*, see Fig. 7 (right), showed that hospital X appeared to have inserted ‘1’ (corresponding to myocardial infarction ‘MI’) for all patients with a positive PCR test. Since it is extremely unlikely that one hospital had almost 2,000 COVID-19 patients exclusively with a history of ‘MI’, it strongly suggests that this is a data entry error. And if not, it would introduce a huge bias towards that hospital. Therefore, all entries at hospital X have been removed (set to ‘NaN’). Moreover, we decided to change it to a more simple ‘Yes’/‘No’/‘Unknown’ schema, since there were only 560 values for ‘angina’ (127), ‘stroke’ (197), ‘MI’ (156) and ‘multiple’ (80) combined (when discarding hospital X). This is a low number to allow any meaningful analyses and only likely to introduce bias and over-fitting towards certain hospitals.

#### PMH Hypertension

In comparison to the original cleaning pipeline we retained the missing values in categorical features that were filled in the raw data as ‘Unknown’ rather than adding it to the ‘NaN’ category. This allows the user to decide themselves to either keep it as additional information or not, as it may influence how these values are handled. Moreover, it has to be noted that the demographics of these two groups differ (as stated in the results section). This might be because patients who did not know or remember their past medical history are more likely to be older and have more comorbid conditions, or they may have been too unwell at presentation to provide a history if suffering from impaired consciousness. It may also be that ‘NaN’ values are more likely to represent ‘no disease’ compared to the ‘Unknown’ values because the importance of a ‘0’ value was not realized and it saved time to not enter any value. This issue affected all categorical features.

### Dates

**Fig. 8:**
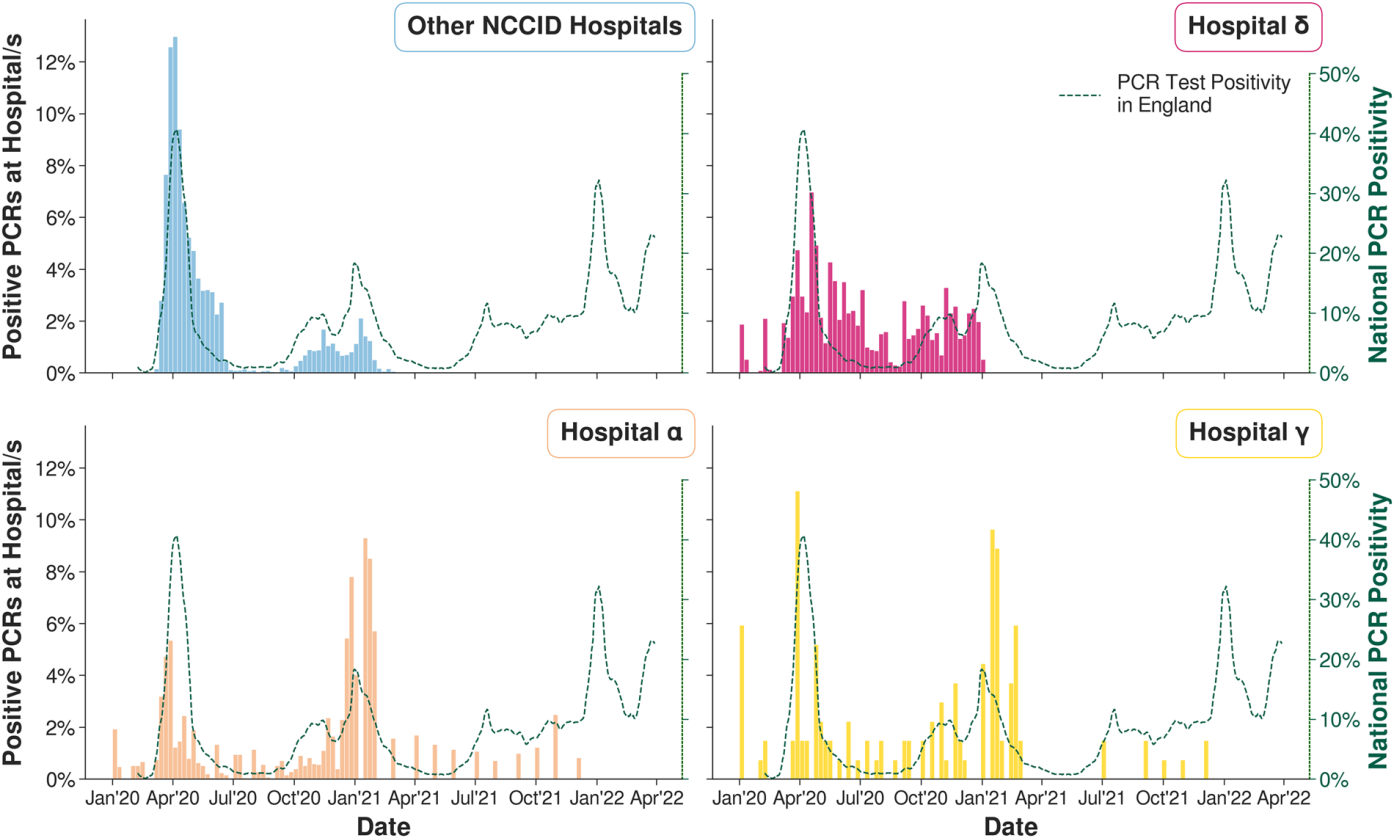
Distribution of dates corresponding to positive RT-PCR results.

The NCCID documentation specifies which date features should be collected in UK and US date format; however, we found that this was entered inconsistently for some hospitals. When importing the raw clinical data with the date manipulation package *pandas*^30,31^ in a Python script, errors were raised for several date entries in diverse features. In the NHSx pipeline individual date corrections were then done based on raised errors (e.g. months with a value greater than 12). We extended that approach by systematically investigating the affected hospitals and then correcting by swapping the date format for all date values in the raised features for these hospitals instead of only individual correction of implausible dates. First, we investigated that the affected data showed implausible data sequences, such as death before admission to the hospital, confirming the concern that the data contains systematic date format inconsistencies. Further analyses showed that indeed there are unexpected peaks in the distribution of day values in dates, with the 1st and 4th of a month being far more common. In April 2020 and January 2021 the largest amount of data was collected for the NCCID corresponding to the two major pandemic waves throughout the UK, see Fig. 8. Swapping UK and US date format transfers these dates to the 1st and 4th of a month rather than January and April. We observed that such mistakes happened systematically at 3 hospitals, see Fig. 8, for which the data peak in April 2020 is much smaller when compared to the other NCCID hospitals. A further indicator of this error is that there are peaks in PCR dates which appear even before the first pandemic wave in April 2020 for these hospitals, whilst in 2021 the dates are almost always recorded at the beginning of the month.

The described date error did not result in loss of date values in the NHSx pipeline, but resulted in the inclusion of incorrect dates. However, we noted that there were still many date values unusable when implementing this cleaning pipeline. By examining those excluded, we identified another significant date error due to the use of Excel spreadsheets to submit data. The software encoded some dates in serial number format, where it stores date and time values in terms of the number of days since 1st January 1900 (e.g. 1st January 2020 is stored as the number 43,831). Such numerical values are now converted into date format to ensure this data is not lost.

### Numerical features

#### Creatinine

The standard unit for creatinine is *μ*mol/L^32^ and values beneath 20 are unlikely to be measured in that unit. In particular, values below 0.5 are likely to be reported in mmol/L and therefore have been converted accordingly. On the other hand, values in the interval [0.5, 20] may be errors or reported in mg/dL. Since no common pattern for mg/dL was found and the values did not appear consistent with their urea or PMH of CKD, we applied clipping, i.e. discarded values in that interval, cf. Figure 4. Unfortunately, we encountered a similar issue with urea, another common blood marker. It appears that results in a mixture of units (mmol/L and mg/dL) have been included, however, the typical references ranges overlap (2–7 mmol/L and 6–20 mg/dL) and it was not possible to distinguish which hospitals used which units.

#### Troponin I

Values beneath 40 ng/L for Troponin I correspond to a healthy, ‘normal’ level^33^. For some numerical results, the lower bound of the laboratory technique or equipment may limit the readings and it is common for low results to be reported with a less than value (shown with the ‘<‘ operator)^34^. For *Troponin I*, we observed that non-numeric entries of ‘<3’, ‘<5’ and ‘<10’ were turned to ‘NaN’ by the NHSx pipeline. We decided to extract these values and truncated them to 10 ng/L, ensuring consistency throughout all of the NCCID laboratories and hospitals. Given that affected values are comfortably within the ‘normal’ range, the result is clinically valuable but the precise value below 10 ng/L has no tangible significance in practice^33^. In addition, having a consistent minima will avoid bias associated with varying detection limits specific to certain hospitals.

#### D-dimer

**Fig. 9:**
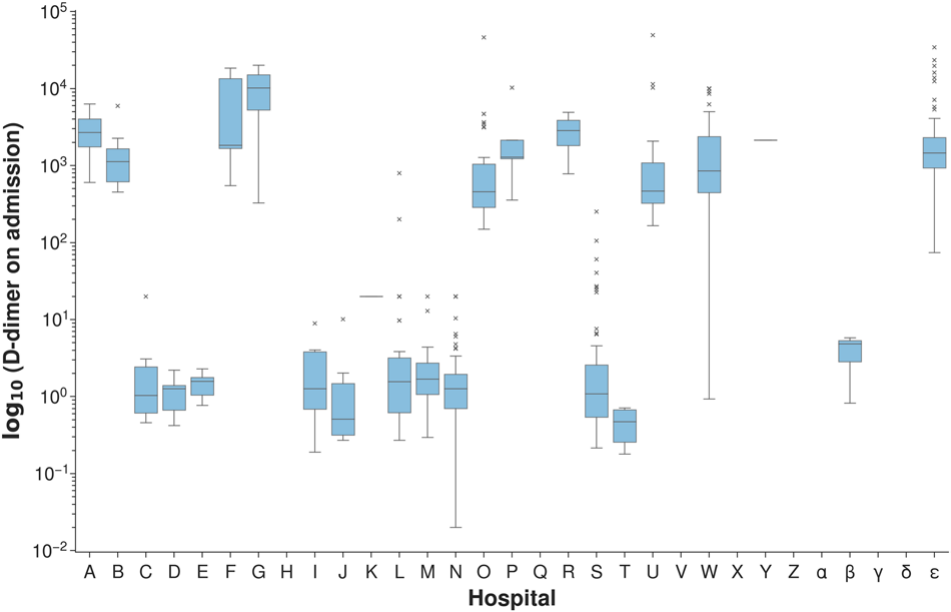
Values for *D-dimer* obtained after employing the NHSx cleaning pipeline.

During attempts to identify reliable prognostic indicators for COVID-19, D-dimer is often cited as an important marker. However, several problems in medical literature have been identified in the reporting of D-dimer results^35^. Firstly, values may be recorded in D-dimer Units (DDU) or Fibrinogen Equivalent Units (FEU). In addition, some hospitals inserted values orders of magnitude apart, as institutions use differing units such as ng/mL, *μ*g/mL, mg/L, g/L (see Fig. 9 demonstrating the obvious inconsistencies). For all of the hospitals in the development data, the units were confirmed by telephone and all values converted to ng/mL FEU, see Fig. 4. Moreover, in order to reduce bias, truncation was applied to reported minimum and maximum values at some hospitals (150 and 10,000 ng/mL FEU).

#### FiO_2_

In the original cleaning pipeline, *FiO*_2_ values up to 15 were remapped from decimals or “liters” to oxygen percentages (%), see Fig. 4, and values representing Venturi masks^36^ were remapped. However, the relationship between “liters of oxygen” and *FiO*_2_ is dependent on a number of factors including: respiratory rate, tidal volume, ventilatory pattern, and oxygen delivery device. It can only serve as an approximation and although combining them may reduce the risk of bias (for example, acting as a proxy allowing the model identify certain centers or types of patient), it may result in errors. With this in mind, the user can choose not to apply this remapping when running the extended pipeline. Furthermore, the user should consider that FiO_2_ is only accurate at 21% (room air) or in a closed system, e.g. a mechanically ventilated patient. In patients receiving supplementary oxygen via “non-closed” systems, FiO_2_ can only be approximated. In addition to remapping *FiO*_2_ units, we set the minimum threshold to 21% (room air) rather than 0%, which would suggest that the patient is breathing no oxygen at all. Therefore, the values are truncated to 21% and clipped at 100%.

#### PaO_2_

The NCCID schema requested the partial pressure of oxygen (PaO_2_) rather than the more commonly collected oxygen saturation (SpO_2_)^23^. SpO_2_, measured by a pulse oximetry, is an approximation of PaO_2_, which avoids the need for a painful and time-consuming arterial blood test. SpO_2_ is typically collected with every set of observations, whereas a PaO_2_ tends to only be performed on deteriorating or acutely unwell respiratory patients. However, both tests may appear within the hospital patient records and as a consequence, there appears to have been some confusion with a mixture of PaO_2_ and SpO_2_ values submitted to the NCCID. Fortunately, these were mostly consistent for each hospital and could be traced back due to the different units and reference ranges, see Fig. 5. For example, results greater than 50 are more likely SpO_2_ values than PaO_2_. In addition, values between [0.5, 1] would be extremely low for a PaO_2_ and most likely represent decimal oxygen saturation. Since SpO_2_ is used more commonly we convert all values to percentage SpO_2_ values. In addition, if a common value of supplementary oxygen was reported, which was non-decimal and less than 50, it was assumed to be *FiO*_2_ entered by mistake and denoted as ‘NaN’. For decimal values between 1 and 50, it was determined to be a correctly reported *PaO*_2_ value and therefore a corresponding value of *SpO*_2_ was imputed via the suggested formula in Gadrey *et al*.^23^ which was developed for non-intubated acute care patients:

$$Pa{O}_{2}={\left(\frac{23,400}{\frac{1}{Sp{O}_{2}}-0.99}\right)}^{\frac{1}{3}}\quad \quad Sp{O}_{2}={\left(\frac{23,400}{Pa{O}_{2}^{3}}+0.99\right)}^{-1}$$

By default, the pipeline returns the feature ‘*SpO2_imputed*’ with missing values imputed from *PaO2* values where possible. However, the settings can be changed to return the data split into two features, *SpO2* and *PaO2*, without imputation and in addition, a column of ‘*PaO2_imputed*’ values can be obtained with missing values imputed from the available *SpO2* data. These options were included to allow the user full autonomy, as other imputation techniques could be considered.

### Sense Checking and Inferences

#### Sense checking

Given that the stated aim is to update NCCID weekly, new data and hospitals will be added, or have already been added, after the development of this pipeline. With this in mind, tools to provide additional sense checks have been implemented that will warn users to closer inspect identified new hospitals or outlying data. Examples of potential errors which are flagged include: *Date of death* occurring before *Date of admission* or *Date of scan*; Lymphocyte count which is greater than their white cell count (WCC); *Date of Intensive Therapy Unit (ITU) admission* is provided but *ITU Admission* is ‘0’. These exceptions are recorded in an additional.csv file to facilitate review and a Jupyter notebook is provided allowing for flagged patients to be reviewed one-by-one if desired.

In addition to the cleaning pipeline, tools for automated exploratory data analysis have been provided which use the ‘DataPrep’ package^20^ in Python. It produces histograms and basic statistics for all features, both for the entire dataset and split by hospital, allowing users to quickly identify additional outliers.

#### Further automated corrections

Throughout the clinical data, there are fields in which it is possible to infer values which have not been completed by the centers. Optional tools have been provided to complete these missing values where possible, for example:

- If *Date of Death* is provided, but *Death* is missing, *Death* is set to ‘1’.
- If there is a *Date Last Known Alive* but *Death* and *Date of Death* are missing, *Death* is set to ‘0’.
- If a *Date of Intensive Therapy Unit (ITU) Admission* is provided, but *ITU Admission* is missing, the latter is set to ‘1’.
- If they have a *Stage of Chronic Kidney Disease (CKD)* but *PMH CKD* is missing, *PMH CKD* is set to ‘1’.

## Data Availability

The NCCID training data is available upon request to users (including software vendors, academics, and clinicians) via a Data Access Request (DAR) process. The following documentation must be completed and signed to apply for access, all of which are available on the NCCID website: a NCCID Data Access Request Form, a Data Access Framework Contract, and a Data Access Agreement. The documents should be returned to *imaging@nhsx.nhs.uk* with further information provided on the website. All of our results may be reproduced using our python package outlined in Section 6. However, as the NCCID data is regularly updated and supplemented, any NCCID files dated after 3rd November 2022 should be excluded in order to fully replicate our findings. The data used in Fig. 8 is available online at https://coronavirus.data.gov.uk and is automatically downloaded by our cleaning package using the UK Government’s ‘Open Data API’. The legal basis for the NCCID is provided in the original article^1^. Ethical approval for this project was originally obtained in April 2020, with substantial amendments approved on 9th August 2021. Approval was provided by the London - Brent Research Ethics Committee, and the Health Research Authority (HRA) and Health and Care Research Wales (HCRW) (IRAS ID: 282705, REC No.: 20/HRA/2504, R&D No.: A095585). The study was performed in accordance with the spirit and the letter of the declaration of Helsinki, the conditions and principles of ICH-GCP, the protocol and applicable local regulatory requirements and laws. Informed consent was not required as data was pseudo-anonymised prior to use by the NHSx AI Lab.

## Code availability

Our extended cleaning pipeline *nccidxclean* is publicly available and can be accessed on GitLab: https://gitlab.developers.cam.ac.uk/maths/cia/covid-19-projects/nccidxclean with accompanying documentation available on the project website (package version at the time of publication: v1.0). The package will automatically download the necessary packages and requirements during installation, including the original NCCID cleaning pipeline upon which our pipeline builds. The python package is independent of the operating system and allows replication of our results using the command line interface, python scripts, or the included Jupyter notebooks. For full analysis, the *return_cols* parameter can be set to ‘all_with_original’, returning all possible data features. Additional code is provided to allow for the original NHSx pipeline to be run using a single command in the command line. The data from the NHSx pipeline may be then used in the analysis subpackage to generate all figures and numerical results found in this work. An additional subpackage *eda* is included for exploratory data analysis of the final cleaned data. Full step-by-step guidance and further details of the package are provided on the project’s website. *Please note that pertinent links related to data and code availability, referenced in Sections 5 and 6, are provided at their initial mention within the main body of the text*.
